# Impact of an integrated parenting and nutrition intervention on growth and development in stunted children at 24 months: evidence from the Stepping Stones programme in rural India

**DOI:** 10.1136/bmjgh-2024-017395

**Published:** 2026-01-08

**Authors:** Abhay Gaidhane, Shital Telrandhe, Penny Holding, Mahalaqua Nazli Khatib, Manoj Patil, Shilpa Gaidhane, Sonali G Choudhari, Roshan Umate, Deepak Saxena, Zahiruddin Quazi Syed

**Affiliations:** 1 Department of Community Medicine, Jawaharlal Nehru Medical College, Wardha; Global Health Academy of Nuvance Health, Danbury, CT, USA; Global Consortium Public Health Research, UK; Datta Meghe Institute of Higher Education and Research, Wardha, Maharashtra, India; 2 Centre of Early Childhood Development – Stepping Stones Project, Research and Development, Datta Meghe Institute of Higher Education and Research, Wardha, Maharashtra, India; 3 Centre of Early Childhood Development - Stepping Stones Project, Research and Develoment, Datta Meghe Institute of Higher Education and Research, Wardha, Maharashtra, India; 4 Department of Physiology, Jawaharlal Nehru Medical College, Wardha; Global Evidence Synthesis Initiative; Division of Evidence Synthesis, School of Epidemiology & Public Health, Datta Meghe Institute of Higher Education and Research, Wardha, Maharashtra, India; 5 Research and Development, Datta Meghe Institute of Higher Education and Research, Wardha, Maharashtra, India; 6 Department of Medicine, Jawaharlal Nehru Medical College, Datta Meghe Institute of Higher Education and Research, Wardha, Maharashtra, India; 7 Department of Community Medicine, Jawaharlal Nehru Medical College, Datta Meghe Institute of Higher Education and Research, Wardha, Maharashtra, India; 8 Centre of Early Childhood Development - Stepping Stones Project, Research and Development, Datta Meghe Institute of Higher Education and Research, Wardha, Maharashtra, India; 9 Indian Institute of Public Health, Gandhinagar, Gujarat, India; 10 Department of Community Medicine, Jawaharlal Nehru Medical College, Wardha; South Asia Infant Feeding Research Network (SAIFRN), Global Health Academy of Nuvance Health, Danbury, USA; ITE Research Foundation, Wardha; School of Epidemiology and Public Health; Datta Meghe Institute of Higher Education and Research, Wardha, Maharashtra, India

**Keywords:** Stunting, Public Health, Child health, Nutrition

## Abstract

**Objectives:**

This paper examines the differential impact of responsive parenting and nutrition interventions on early child development in stunted versus never-stunted children at 24 months of age in rural India.

**Methods:**

We conducted a secondary analysis of data from Stepping Stones—a cluster randomised controlled trial involving 21 subcentres/clusters allocated to intervention or control group. The intervention comprised home visits, group sessions and community workshops, focusing on responsive parenting and nutrition. Developmental outcomes, cognitive, motor, language and socio-emotional, were assessed at 24 months using validated tools. A mixed-effects regression model with an interaction term was used to estimate effect sizes for stunted and never-stunted children.

**Results:**

Among 588 children analysed, 35.9% were stunted at 24 months, and 35 (5.95%) of them exhibited ‘early-onset persistent stunting’. The intervention improved cognitive (β=0.22), motor (β=0.23), language (β=0.17) and socio-emotional development (β=0.23) in never-stunted children compared with those who have not received intervention. For stunted children who have not received the intervention, a development score was lower for all development domains compared with never-stunted children, but not statistically significant (p>0.05). Although the effects differed between stunted and never-stunted children, the interaction effects between intervention and stunting were not statistically significant across all domains (p>0.05), indicating that the intervention’s benefits did not significantly differ by stunting status.

**Conclusion:**

Integrated parenting and nutrition interventions improved developmental outcomes across all domains, regardless of the child’s stunting status. These findings support universal application of such programmes, highlighting the need to integrate them into existing child development and nutrition programmes.

**WHAT IS ALREADY KNOWN ON THIS TOPIC**

- Stunting, driven by a combination of genetic, nutritional and environmental factors, is a well-established marker of developmental risk and is closely linked to poverty in low- and middle-income countries (LMICs).
- Children who are stunted are more likely to experience delays in cognitive and socio-emotional development, which can negatively affect schooling, future earnings and overall productivity.
- Prior research has shown that responsive caregiving and adequate nutrition are critical for improving early childhood development (ECD). However, there is limited evidence on whether the impact of parenting and nutrition interventions differs between stunted and never-stunted children. Understanding these differential effects is particularly important in LMICs, where stunting is highly prevalent and developmental vulnerabilities are widespread.

**WHAT THIS STUDY ADDS**

- This study provides new evidence on the impact of integrated responsive parenting and nutrition interventions on ECD outcomes—cognitive, motor, language and socio-emotional development at 24 months of age in rural India in both stunted and never-stunted children.
- Importantly, the benefits of the intervention, regardless of stunting status, highlight the value of universal application of parenting and nutrition programmes.
- These findings fill an important gap in the literature by showing that integrated ECD interventions can support developmental gains across diverse nutritional subgroups in low-resource settings.

**HOW THIS STUDY MIGHT AFFECT RESEARCH, PRACTICE OR POLICY**

- This study highlights the need for further research to explore how parenting and nutrition interventions influence developmental outcomes across different child age groups, settings and cultural contexts in stunted and non-stunted children. It underscores the importance of evaluating the long-term impacts of integrated interventions, particularly in LMICs where stunting remains widespread. The findings also point to the value of conducting larger, adequately powered studies to detect subgroup effects, thereby informing the design of more targeted and equitable ECD strategies.
- This study reinforces that responsive parenting and nutrition interventions improve early childhood development outcomes regardless of a child’s stunting status. By demonstrating broad developmental benefits, the study provides evidence for programme implementers to integrate responsive caregiving and nutritional support into routine child health and nutrition services, particularly in settings where undernutrition and developmental delays often coexist.
- This study makes a strong case for the universal integration of parenting and nutrition programmes into national health and development strategies, particularly in contexts where stunting remains widespread. The findings suggest that interventions reaching both stunted and non-stunted children can play a crucial role in promoting equitable and optimal developmental outcomes at the population level, particularly in low-income and middle-income settings where developmental risks are widespread.

## Introduction

 Stunting in children results from a combination of factors, including genetics, nutrition, psychosocial influences and environmental conditions. As per WHO, children are defined as stunted if their height-for-age is more than two SD below the WHO Child Growth Standards median.^1^ It serves as a clear indicator that children are facing developmental challenges, reflecting both past deprivation and forecasts for potential impact on future life.^2^ Stunting exacerbates intergenerational poverty because it disproportionately affects poor and marginalised populations, particularly from the low- and middle-income countries (LMICs).^3^,^5^ Globally, in 2022, 149 million children under 5 were estimated to be stunted,^6^ and in India, nearly 35.5% of children under age 5 years are stunted.^7^

Studies have reported that cognitive and behavioural development is compromised in stunted children, which may be attributed to the causal relation between stunting and early childhood development (ECD),^8 9^ or both the stunting and ECD outcomes share common social determinants, like poverty, income and caregivers’ education.^5 8 10^ Studies have also shown that maternal and child malnutrition, as well as poor cognitive, social and emotional development in the early years, impacts development and functionality, leading to impaired cognitive abilities, poorer educational outcomes, lower wages and decreased productivity in later life.^11^,^14^

Research has highlighted the importance of empowering parents and caregivers to provide comprehensive, nurturing care, including psychosocial stimulation, proper nutrition and emotional support for children’s holistic growth and development.^15^,^18^ The effectiveness of such intervention is influenced by contextual factors such as societal, cultural and economic determinants and the availability of healthcare and nutrition services.^14^,^17^

Earlier studies have not sufficiently explored whether parenting and nutrition interventions have differing effects on stunted versus never-stunted children. Given the high rates of stunting in LMICs, it is essential to understand how integrated, responsive parenting and nutrition interventions impact ECD in both stunted and never-stunted children at 24 months of age, particularly in rural, low-resource settings.

This paper examines the data from a previously conducted cluster-randomised controlled trial (cRCT) to study the effect of responsive parenting and nutrition programmes on the development of 2-year-old children.^19^ We compared ECD outcomes in stunted children with those who were never stunted to understand the differences in the programme’s effectiveness between the two groups.

## Methods

### Settings and context

The initial trial was conducted in a remote rural area near a forest in two districts of central India. This area is economically underprivileged, with most people relying on farming, forest produce and livestock for their livelihood. Access to health services and social services is challenging for the residents. Sociocultural beliefs and practices affecting health are prevalent in the study area. The Anganwadi Centers (AWC), under the Integrated Child Development Scheme (ICDS), offer informal education to children between 3 and 6 years and nutritional support to children under five. Each AWC, managed by the Anganwadi workers (AWWs), serves about 1000 population. Children receive non-formal education, dietary supplements and growth monitoring in AWC.

For this study, we analysed data from our Stepping Stones programme. The Stepping Stones programme aimed to positively impact the motor, cognitive, language development and socio-emotional skills and physical growth of young children under 2 years through responsive parenting and nutrition interventions.^19^ This was achieved by enhancing the capacity of caregivers for responsive parenting and nutritional practices. The intervention was delivered through home visits and group sessions. Balsakhi, a community volunteer, delivered the Stepping Stones programme, with support from AWWs in the respective village. This paper examines the impact of this combined intervention on the development of children who were stunted at 24 months of age compared with children who were never stunted. The primary study was implemented in central India’s Seloo and Hingana blocks.

### Study design

The main study was a cRCT, with randomisation conducted at a subcentre level. A subcentre is the lowest-level health facility in the public health system in India, typically covering a population of 5000 to 6000 in rural areas.

The nature of the intervention—promoting ECD through parenting sessions, group meetings and home visits—created a high risk of contamination if individuals within the same geographic or social unit were allocated to different groups. Randomising entire sub-centre catchment areas reduced this risk and preserved the integrity of the intervention and control conditions. Participants from the intervention clusters received responsive parenting and nutrition interventions and standard care through AWC. The control group received only the existing standard services through AWC.

### Randomisation and masking

Randomisation was conducted at the subcentre level, which served as a unit of allocation, to minimise contact between intervention and control groups, reducing the risk of intervention dilution. We used stratified randomisation to ensure balanced allocation of subcentres (clusters) to the intervention and control groups across geographic and administrative units. The 21 eligible subcentres were identified from four primary health centres (PHCs), each serving as a stratum to account for potential differences in population characteristics and health system functioning. Within each PHC stratum, subcentres were randomised in a 1:1 ratio to intervention or control groups using a computer-generated random number table. The randomisation was conducted by an independent statistician who was not involved in intervention implementation or outcome assessment, ensuring allocation concealment. In total, 11 subcentres (covering 58 villages) were assigned to the intervention group and 10 subcentres (covering 48 villages) to the control group.

### Study participants

Pregnant women in their first or second trimester, permanent residents of the study area were included. High-risk pregnancies (eg, severe anaemia, gestational diabetes, hypertension, previous pregnancy complications or chronic illnesses requiring specialised care) were excluded to reduce confounding and ensure participant safety. The enumerators, who recruited the pregnant mothers, were blinded to the allocation of the intervention group and the control group. Recruitment of the study participants was completed in 6 months, and the study cohort was followed until their children reached the age of 2. Pregnant women and caregivers were counselled, explaining the objectives and need for the study. Informed consent was taken from the study participants. The study team held awareness meetings with community members, AWWs and local leaders to gain their support for the study intervention.

### Registration

The trial was registered at the Clinical Trial Registry of India under the CTRI Number: CTRI/2017/05/008553 on 15 May 2017.

### Intervention

An integrated parenting and nutrition programme was introduced to enhance caregivers’ knowledge, skills and behaviour across six key areas of parenting: stimulation, nutrition, physical activities, shelter, safety, and protection and discipline, as outlined by O’Donnell *et al*.^20^ We hypothesise that strengthening caregivers’ knowledge, skills and practices in these areas will foster a nurturing household environment, leading to significant improvements in child development.^21^

### Programme delivery

The programme was implemented by ‘Balsakhis’, local community volunteers with a high school education, who were committed to volunteering 2 hours daily. Each village had one Balsakhi, supported by an AWW. Implementation included biweekly home visits, monthly group sessions and community workshops focusing on toy/play material development, nutritional demonstrations, locally relevant recipes and community awareness meetings over a period of 24 months. Balsakhis and AWWs received 3 days of certified training to deliver the integrated intervention, which included parenting and nutrition support through fortnightly home visits, group sessions and community workshops. The training curriculum and methods were standardised and delivered by trained facilitators using a participatory approach. To ensure the fidelity of delivery, field supervisors and AWWs accompanied Balsakhis on home visits. The intervention delivery was monitored through regular supervision, caregiver feedback and periodic evaluations. Balsakhis received compensation for expenses and refresher training as needed.

The home visit curriculum aimed to improve caregiving skills in six areas of parental competencies, namely (1) shelter and nurturing care, (2) food and nutrition, (3) protection and discipline, (4) socio-emotional learning, (5) health, and (6) early childhood education through 44 visits over 24 months, using a reflective approach and a tool kit comprising various educational and play materials. Community workshops were held in each study village, with six sessions per village, focusing on creating and using ECD toys from local materials. Nutrition support included establishing four nutrition demonstration centres and 18 household nutrition gardens. We linked beneficiaries to the ICDS services for nutrition supplementation. The Monthly Community Group Meetings facilitated peer learning and provided a platform for caregivers to discuss childcare challenges and share experiences.^19 21^

### Monitoring, evaluation and learning (MEL)

We developed a robust MEL system that is dynamic, inclusive, community-centred and informative. The team conducted weekly data reviews to ensure comprehensive coverage and maintain the fidelity of the intervention. This involved tracking the frequency of service utilisation and the level of engagement during interactions. We also closely monitored the participation of mothers/caregivers and male members from the household in home visits and group sessions, and adherence to the scheduled timeline for home visits. Programme supervisors oversaw activities in the field, providing real-time feedback and training to Balsakhis. The details of the MEL approach and its impact on coverage and quality of intervention were presented in a paper by Gaidhane *et al*.^21 22^

### Outcome and assessments

The study’s primary outcomes were four separate domains of child development assessed at 24 months—cognitive, language, socio-emotional development and motor skills. Anthropometric data related to stunting, wasting and undernutrition were collected at birth and then again at 6, 12, 18 and 24 months. Child development outcomes were assessed at 12 and 24 months using a comprehensive set of tools previously validated in LMICs. Motor, language, cognitive and personal-social development were assessed using the Developmental Milestones Checklist.^23 24^ Social and emotional development was assessed using the Profile of Socio-Emotional Development tool.^25^ The home environment was evaluated at baseline and at endpoint using the Infant-Toddler Home Inventory,^26^ while the mother-child interaction was assessed using the Observation of the Mother-Child Interaction tool.^27^ All the tools were adapted to the local context and translated into Marathi and validated through backtranslation into English. Independent, trained and certified assessors, blinded to group assignments, conducted all assessments to maintain objectivity.

We conducted anthropometric assessments following WHO protocols^28^ using calibrated and certified tools to ensure accuracy. Outcome assessors were trained on anthropometric assessment protocols, and the data manager periodically reviewed their data for quality and completeness.

### Analysis

#### Sample size

This secondary analysis considered the full sample from the main trial. The original trial was powered to detect a minimum difference of 0.5 SD in child development outcomes between the intervention and control groups. The effect size of 0.5 SD was based on prior ECD studies in LMICs, where effects between 0.3 and 0.6 SD have been reported. A 0.5 SD difference is widely accepted as a moderate and meaningful effect in child development trials, reflecting both clinical relevance and policy significance in our context.^29 30^

We based our calculations on an intra-cluster correlation coefficient of 0.02, an average of 30 pregnancies per cluster per year and a design effect of 1.58. With these assumptions, we determined that a sample size of 452 mother and child pairs was needed to achieve 95% confidence and 80% power. Anticipating a 20% loss to follow-up throughout the project duration, we adjusted the final sample size to a minimum of 542 mother and child pairs (271 in each group), eventually distributed across 21 clusters. From an ethical standpoint, we included all eligible children who met the inclusion criteria. Thus, we recruited 656 participants for the study, and of these, 588 were completers. Therefore, our outcome analysis considered a total of 588 participants (296 from the intervention group and 292 from the control group). The Consolidated Standards of Reporting Trials flow diagram of the primary trial is presented in online supplemental Figure S1 for reference.

#### Data analysis

We used Stata V.14 for data analysis. We examined the household and sociodemographic data at recruitment, between the intervention and control groups to verify the robustness of the randomisation process. Comparisons between baseline characteristics of subgroups (eg, stunted vs non-stunted children, or dropouts vs completers) were conducted using appropriate tests of significance, Student’s t-test for continuous variables and the χ^2^ test for categorical variables, accounting for clustering at the subcentre level considering the clustered study design. All baseline comparisons incorporated cluster-adjusted SEs at the subcentre (village) level using robust variance estimators, thereby correcting test statistics and corresponding p values for intra-cluster correlation.

The main analyses focused on the differential effect of the intervention on cognitive, language, motor and socio-emotional development outcomes in stunted and never-stunted children. For the main outcomes, we calculated mean scores with 95% CIs for cognitive, language, motor and socio-emotional development, separately for the overall sample, stunted children and never-stunted children. Raw developmental scores were converted into age-standardised Z-scores using the sample’s own means and SD. Anthropometric measurements were converted to Z-scores based on WHO growth standards; stunting was defined as a height-for-age Z-score below −2 SD.

To assess the impact of the intervention, the core outcome variable outcomes—cognitive, language, motor and socio-emotional development—were modelled separately using mixed-effects maximum likelihood regression models with an interaction term between intervention status and stunting status. This allowed us to examine the differential effects of the intervention in stunted versus never-stunted children. The models included intervention groups and stunting status as fixed effects. Cluster (subcentre) and assessors included as random effects to adjust for the intra-cluster correlation and potential variability introduced by different assessors since assessors conducted assessments across multiple clusters. This approach avoids underestimation of SEs and improves the precision of effect estimates. We adjusted for potential confounders, including the child’s age and sex, maternal age and education, family size, household tobacco use and household wealth index.

We did not include cluster-level characteristics such as PHC catchment size and service availability in the regression models, as these factors were largely homogeneous across clusters. The study area had comparable levels of primary healthcare access and service delivery across subcentres, and stratified randomisation further ensured balance across intervention and control group.

## Results

We conducted 6665 home visits and held 25 community workshops and 450 community meetings throughout the intervention duration. Of the total 588 children, 291 (49.49%) were male and 297 (50.51%) were female. The characteristics of study participants from the intervention and the control group were similar at recruitment (table 1). We also found no significant differences between the participants who dropped out and those who remained in the trial (online supplemental Table S1).

**Table 1:** Baseline characteristics of study participants in the intervention and the control group

	Total (n=656)	Intervention (n=326)	Control (n=330)	P value
Maternal characteristics				
Age in years, mean (SD)	23.94 (3.61)	23.79 (3.57)	24.08 (3.64)	p=0.288
Education				
Illiterate	18 (2.74%)	11 (3.37%)	7 (2.12%)	χ^2^(4)=6.06, p=0.195
Primary (1–5)	24 (3.66%)	12 (3.68%)	12 (3.64%)	
Secondary (6–10)	295 (44.97%)	156 (47.85%)	139 (42.12%)	
Higher secondary	203 (30.95%)	100 (30.67%)	103 (31.21%)	
Graduate and more	116 (17.68%)	47 (14.42%)	69 (20.91%)	
Pregnancy stage at recruitment				
First trimester	132 (20.12%)	68 (20.86%)	64 (19.39%)	χ^2^(1)=0.21, p=0.64
Second trimester	524 (79.88%)	258 (79.14%)	266 (80.61%)	
Gravida				
First	182 (42.99%)	150 (46.01%)	132 (40%)	χ^2^(3)=5.68, p=0.224
Second	294 (44.82%)	145 (44.48%)	149 (45.15%)	
Third	64 (9.76%)	26 (7.98%)	38 (11.52%)	
Fourth and above	16 (2.44%)	5 (1.54%)	11 (3.34%)	
Total of live children, mean (SD)	0.59 (0.65)	0.61 (0.68)	0.58 (0.65)	p=0.576
Anaemia				
No anaemia	173 (30.40%)	82 (29.82%)	91 (30.95%)	χ^2^(3)=2.43, p=0.487
Mild anaemia	203 (35.68%)	96 (34.91%)	107 (36.39%)	
Moderate anaemia	191 (33.57%)	95 (34.55%)	96 (32.65%)	
Severe anaemia	2 (0.35%)	2 (0.73%)	0	
Father’s characteristics				
Age in years, mean (SD)	29.99 (4.13)	29.59 (3.63)	30.4 (4.54)	p=0.012
Education				
Illiterate	21 (3.20%)	13 (3.99%)	8 (2.42%)	χ^2^(4)=4.67, p=0.322
Primary (1–5)	47 (7.16%)	23 (7.06%)	24 (7.27%)	
Secondary (6–10)	337 (51.37%)	175 (53.68%)	162 (49.09%)	
Higher secondary	170 (25.91%)	82 (25.15%)	88 (26.67%)	
Graduate	81 (12.35%)	33 (10.12%)	48 (14.55%)	
Household characteristics				
Caste category				
Schedule caste	52 (8.84%)	24 (8.11%)	28 (9.59%)	χ^2^(3)=14.59, p=0.002
Schedule tribe	235 (39.97%)	141 (47.64%)	94 (32.19%)	
Backward classes	279 (47.45%)	123 (41.56%)	156 (53.42%)	
Open/general	22 (3.74%)	8 (2.70%)	14 (4.79%)	
Wealth index				
1 - Lowest	110 (16.77%)	49 (15.03%)	61 (18.48%)	χ^2^(4)=7.809, p=0.099
2 - Second	122 (18.60%)	58 (17.79%)	64 (19.39%)	
3 - Third	143 (21.80%)	83 (25.46%)	60 (18.18%)	
4 - Fourth	142 (21.65%)	75 (23.01%)	67 (20.30%)	
5 - Fifth	139 (21.19%)	61 (18.71%)	78 (23.64%)	
Average family size, mean (SD)	4.66 (1.84)	4.86 (1.91)	4.47 (1.76)	p=0.006
Below poverty line	286 (43.66%)	145 (44.62%)	141 (42.73%)	χ^2^(1)=0.03, p=0.626

The df for continuous variables was based on the number of clusters and number of groups.

Table adapted from Gaidhane *et al*.^19^

Data are No. (%), unless stated otherwise.

Table 2 presents the household characteristics of the stunted and never-stunted children. Most of the stunted children belong to other backward caste categories, followed by the scheduled tribes. Nearly 67% of the stunted children belong to wealth quantiles three or below compared with 53% of children who were never stunted (p<0.01). The average family member in the stunted group was less than the never-stunted children, and nearly one-third of the stunted children belonged to the below poverty line (table 2).

**Table 2:** Household characteristics of stunted and never-stunted children

Variable	Overall, n=588	Stunted, n=211	Never-stunted, n=377	P value
Caste, No. (%)				0.670
Open/general	21 (3.57)	7 (3.32)	15 (3.97)	
Other backward classes	280 (47.61)	94 (44.55)	185 (49.07)	
Scheduled caste	52 (8.84)	20 (9.48)	32 (8.48)	
Scheduled tribe	235 (39.96)	90 (42.65)	145 (38.36)	
Wealth quintile, No. (%)				0.003
1—lowest	94 (15.96)	42 (19.91)	52 (13.76)	
2—second	113 (19.19)	51 (24.17)	62 (16.40)	
3—middle	126 (21.39)	47 (22.27)	79 (20.90)	
4—fourth	125 (21.22)	39 (18.48)	86 (22.75)	
5—highest	131 (22.24)	32 (15.17)	99 (26.19)	
Total family members, mean (SD)	4.69 (1.85)	4.55 (1.70)	4.77 (1.93)	0.166
Below poverty line, No. (%)	256 (43.54)	100 (47.39)	156 (41.38)	0.158
Card issued, No. (%)				0.029
Yellow ration card	168 (28.57)	64 (30.33)	104 (27.58)	
Saffron ration card	358 (60.88)	118 (55.92)	240 (63.66)	
White ration card	10 (1.70)	2 (0.95)	8 (2.12)	
Not issued	52 (8.84)	27 (12.80)	25 (6.63)	
Kitchen separate, No. (%)	526 (89.45)	188 (89.10)	338 (89.65)	0.905
House ownership, No. (%)				0.187
* *Own	547 (93.02)	192 (91.00)	355 (94.16)	
* *Rent	42 (7.14)	19 (9.00)	23 (6.10)	

Table adapted from Gaidhane *et al*.^19^

As per the definition of stunting, out of 588 children recruited in the trial, 211 (35.88%) were stunted at 24 months. 35 (5.94%) exhibited ‘early-onset persistent stunting’, meaning the stunting began before the child reached 6 months and continued until they were 24 months old. For the remaining 176 (29.88%) stunted children, stunting onset occurred after 6 months but persisted until 24 months. The average Height for Age Z-score (ZHA) (SD) of children at 12 months, 18 months and 24 months was −0.96 (SD 1.31), –1.29 (SD 1.09) and −1.33 (SD 0.99), respectively. In 35 children with ‘early-onset persistent stunting’, the intervention was associated with significant positive effects on cognitive development (coefficient=0.271; 95% CI 0.004 to 0.52), motor development (coefficient=0.27; 95% CI 0.11 to 0.17), language development (coefficient=0.207; 95% CI 0.04 to 0.45) and socio-emotional development (coefficient=0.23; 95% CI 0.05 to 0.41).

Table 3 presents the mean (SD) of development scores for the intervention and control groups in stunted and never-stunted children. For both the never-stunted and stunted children, the intervention consistently demonstrated higher mean developmental scores than those in the control group for all developmental domains. For stunted children, the intervention positively impacted all development domains. However, it was statistically significant for cognitive development (p=0.048). The intervention showed a statistically significant effect on all development domains for never-stunted children.

**Table 3:** Mean score of development domains in the intervention and the control group for all children, stunted children and never-stunted children

Developmental domains	All children (n=588)		Stunted (n=211)		Never-stunted (n=377)	
	Intervention (n=296)	Control (n=292)	Intervention (n=108)	Control (n=103)	Intervention (n=188)	Control (n=189)
Cognitive	71.62 (8.55)	69.38 (9.99)	70.58 (9.72)*	67 (10.47)	72.22 (7.77)*	70.69 (9.49)
Language	18.25 (4.86)	17.41 (5.35)	17.91 (5.02)	16.38 (5.13)	18.46 (4.77)*	17.97 (5.40)
Socio-emotional	21.48 (5.21)	20.51 (5.62)	20.84 (5.20)	19.95 (6.11)	21.85 (5.19)*	20.82 (5.33)
Motor	53.36 (5.21)	51.97 (6.01)	52.67 (6.34)	50.61 (6.62)	53.76 (4.41)*	52.72 (5.52)

Values are presented as mean (SD)*.*

* Indicates statistically significant difference (p<0.05) between the intervention and control groups within the same category (ie, all children, stunted, or never-stunted).

Table 4 shows the impact of the intervention and stunting on developmental outcomes, highlighting the differences in effect between stunted and non-stunted children. The effect size is reported as a standardised coefficient in SD units, with a 95% CI.

**Table 4:** Effect of intervention and stunting on development outcomes at 24 months of age

Parameters		Standardised estimates* (95% CI)	P value
Cognitive development	Intervention	0.22 (0.22 to 0.43)	0.031
	Stunting	−0.26 (−0.49 to 0.02)	0.033
	Intervention × Stunting†	0.14 (−0.19 to 0.46)	0.413
Motor development	Intervention	0.23 (0.03 to 0.43)	0.022
	Stunting	−0.25 (−0.49 to −0.01)	0.039
	Intervention × Stunting†	0.07 (−0.25 to 0.41)	0.655
Language development	Intervention	0.17 (−0.05 to 0.40)	0.132
	Stunting	−0.19 (−0.42 to 0.04)	0.115
	Intervention × Stunting†	0.16 (−0.17 to 0.48)	0.342
Socio-emotional development	Intervention	0.23 (0.01 to 0.44)	0.037
	Stunting	−0.13 (−0.38 to 0.10)	0.266
	Intervention × Stunting†	0.01 (−0.32 to 0.34)	0.946

The raw scores for all developmental domains were converted into age-standardised Z-scores. We used a mixed-effects maximum likelihood (ML) regression model to estimate the effect sizes. Intervention and stunting status were treated as fixed-effect parameters, while clusters and assessors were modelled as random effects. The analysis was adjusted for covariates, including child age and sex, mother’s age, maternal education, family size, tobacco consumption and wealth index.

* The effect size is expressed as a standardised effect in SD units, along with its 95% CI.

† Intervention and Stunting interaction.

Among the children in the intervention group, those who were never stunted had cognitive development scores that were 0.22 SD higher than children in the control group (p=0.031). In stunted children, cognitive scores were 0.26 SD lower than those of never-stunted children (p=0.03). The interaction effect between intervention status and stunting on cognitive development was 0.14 SD, but this difference was not statistically significant (p=0.413) (table 4).

Among children from the intervention group, those who were never stunted had motor development scores that were 0.23 SD higher than children in the control group (p=0.022). Those who were stunted had motor scores that were 0.25 SD lower than never-stunted children (p=0.039). The interaction effect between intervention status and stunting on motor development was 0.07 SD; however, this was not statistically significant (p=0.655) (table 4).

Among children from the intervention group, those who were never stunted had language development scores that were 0.17 SD higher than children in the control group (p=0.132). Those who were stunted had language scores that were 0.19 SD lower than their never-stunted peers (p=0.115). The interaction effect between intervention status and stunting on language development was 0.16 SD; however, this difference was not statistically significant (p=0.342) (table 4).

Among children from the intervention group, those who were never stunted had socio-emotional development scores that were 0.23 SD higher than children in the control group (p=0.037). Those who were stunted had socio-emotional scores that were 0.13 SD lower than their never-stunted peers, though this difference was not statistically significant (p=0.266). The interaction effect between intervention status and stunting on socio-emotional development was minimal (0.01 SD) and not statistically significant (p=0.947) (table 4).

To examine whether the associations between key covariates and developmental outcomes differed by stunting status, we introduced interaction terms between stunting and selected covariates into the mixed-effects regression models. These covariates included maternal education, household wealth score, maternal age, family size and gender of the child. Table 5 presents the associations between key covariates and developmental outcomes, stratified by stunting status. Maternal education showed a stronger and statistically significant association with cognitive development in never-stunted children (β=0.045, 95% CI 0.007 to 0.08). Among stunted children, maternal education was significantly associated only with language development (β=0.419, 95% CI 0.002 to 0.817), though with wide CIs, therefore the less precise estimates. Wealth score was positively associated with cognitive, motor and language outcomes in both stunted and never-stunted children, with statistically significant interactions across all three domains (p<0.05). No consistent associations were observed for maternal age or family size across groups. Child gender showed a borderline association with cognitive and language development, particularly among never-stunted children (table 5).

**Table 5:** Effects of covariates on developmental domains stratified by stunting status

	Cognitive development			Motor development			Language development			Socio-emotional development		
Covariate	Never-stunted β (95% CI)	Stunted β (95% CI)	P value	Never-stunted β (95% CI)	Stunted β (95% CI)	P value	Never-stunted β (95% CI)	Stunted β (95% CI)	P value	Never-stunted β (95% CI)	Stunted β (95% CI)	P value
Child gender (female)	0.16 (−0.02 to 0.34)	0.104 (−0.18 to 0.38)	0.086	0.090 (−0.018 to 0.03)	0.011 (−0.18 to 0.41)	0.229	0.19 (−0.003 to 0.38)	0.068 (−0.19 to 0.32)	0.074	0.012 (−0.18 to 0.207)	0.193 (−0.071 to 0.457)	0.476
Maternal age in years	−0.005 (−0.03 to 0.01)	−0.003 (−0.04 to 0.035)	0.645	0.006 (−0.018 to 0.030)	0.011 (−0.031 to 0.053)	0.467	−0.018 (−0.04 to 0.008)	−0.019 (−0.055 to 0.015)	0.110	0.006 (−0.021 to 0.033)	0.018 (−0.017 to 0.054)	0.652
Maternal education	0.045 (0.007 to 0.08)	0.029 (−0.01 to 0.07)	0.010	0.015 (−0.021 to 0.052)	0.017 (−0.032 to 0.065)	0.350	0.062 (0.022 to 0.103)	0.419 (0.002 to 0.817)	0.001	0.028 (−0.012 to 0.069)	−0.005 (−0.047 to 0.037)	0.523
Family Size	0.001 (−0.05 to 0.05)	−0.005 (−0.095 to 0.084)	0.904	0.006 (−0.04 to 0.056)	−0.037 (−0.13 to 0.059)	0.833	−0.004 (−0.059 to 0.050)	0.022 (−0.059 to 0.103)	0.930	0.002 (−0.051 to 0.057)	−0.023 (−0.105 to 0.059)	0.677
Wealth Score	0.052 (0.003 to 0.101)	0.089 (0.014 to 0.16)	0.002	0.055 (0.009 to 0.102)	0.089 (0.01 to 0.169)	0.002	0.036 (−0.016 to 0.087)	0.072 (0.005 to 0.139)	0.020	−0.027 (−0.078 to 0.024)	0.0617 (−0.008 to 0.132)	0.737

## Discussion

This study examined the impact of integrated, responsive parenting and nutrition programmes on ECD in stunted and never-stunted children. The results showed significant improvements in all developmental domains for children in the intervention group, particularly among never-stunted children. However, the interaction effects between intervention and stunting were not statistically significant, indicating similar benefits across both groups i.e. stunted and never stunted. Though earlier studies highlighted that physical, mental and emotional stimulation is key to early childhood cognitive development,^8 12 31^ and also highlighted the importance of linear growth during the initial 2 years of life, linking it to immediate and long-term neurodevelopmental outcomes,^3 8 11 15 23 24^ the differential impact of integrated parenting and nutrition programmes on stunted and never-stunted children was inadequately explored. Our study addresses this gap by examining the differential effects of parenting and nutrition interventions on early childhood development, irrespective of children’s linear growth.

While stunting is often considered a barrier to developmental gains due to biological and environmental constraints, our findings suggest that responsive caregiving and nutritional support may benefit children at 24 months of age regardless of stunting status. This aligns with emerging literature suggesting that timely and enriched caregiving environments can mitigate some of the developmental risks associated with chronic undernutrition.^12 13 15 24^ The lack of significant differential effect may reflect the intervention’s ability to reach children early—before developmental delays become entrenched.

The integrated, responsive parenting and nutrition programmes improved cognitive development (0.22 SD units) for never-stunted children but did not significantly differ in their impact between stunted and never-stunted children (p=0.413). Intervention also led to a modest improvement in motor development (0.23 SD units) for never-stunted children, with a minimal and non-significant difference in impact (0.07 SD units) between stunted and non-stunted children. Language development showed a small increase of 0.17 SD units for never-stunted children and a decrease of 0.19 SD units for stunted children without the intervention, with both differences being statistically non-significant (p=0.655). Finally, the intervention improved socio-emotional development (0.23 SD units) in never-stunted children, with negligible and statistically non-significant differences (0.01 SD units) between stunted and never-stunted children (p=0.946). Overall, the intervention’s effects on developmental outcomes did not significantly vary based on stunting status.

This paper also highlights the differential role of socioeconomic factors in shaping developmental outcomes among stunted and never-stunted children. Maternal education and household wealth—two key proxies for nurturing care and resource access—were significantly associated with better cognitive outcomes among never-stunted children, but these associations were non-significant when compared with stunted children. The weaker associations observed in stunted children could be due to persistent biological constraints (eg, early undernutrition), cumulative risk exposure or reduced responsiveness to caregiving inputs. This underscores the importance of integrated early interventions that not only target parenting and stimulation but also address underlying nutritional and structural inequities, especially for children who are biologically and socially disadvantaged.

The Stepping Stones programme combined multiple strategies aligned with the Nurturing Care Framework, which includes regular home visits, play-based stimulation, group meetings and nutrition-focused demonstrations. These activities likely enhanced caregivers’ responsiveness and enriched the child’s environment. The potential pathways for responsive parenting and nutrition programmes to positively impact ECD are nutritional support, stimulation, responsive caregiving, healthcare access and social determinants, each contributing to positive ECD outcomes in never-stunted as well as stunted children.^1015 19 21 25 32^,^34^ Encouraging sensitive responsiveness to a child’s needs, positive reinforcement and consistent discipline are critical, as they may protect against stressors such as poverty or inadequate healthcare, promoting resilience.^35^,^37^ Promoting activities like reading, playing and interactive conversations is critical to stimulating cognitive, linguistic and socio-emotional development.^37^,^41^ Educating caregivers/parents about balanced diets rich in essential nutrients to address nutritional deficiencies and supporting physical growth, brain development, organ function and overall well-being will be critical. Similarly, access to healthcare services for growth monitoring, vaccinations and early intervention will enhance the effectiveness of child development interventions.^42^,^44^

This secondary analysis was not originally powered to detect interaction effects by stunting status, and subgroup analyses may be underpowered. The small number of children with ‘early-onset persistent stunting’ also limited our ability to draw conclusions about this high-risk group. Earlier studies have emphasised that the duration of stunting, especially ‘early-onset persistent stunting’, is crucial to child development.^9 12 24^ Future studies should consider longer-term follow-up and larger samples to explore sustained impacts, particularly in vulnerable subpopulations.

Despite these limitations, our findings provide valuable insights into the impact of integrated parenting and nutrition interventions on children’s developmental outcomes at 24 months of age, irrespective of stunting status. These results carry significant implications for public health and policy. First, they affirm that early parenting interventions are universally beneficial and should be central to child development programmes. The consistent effectiveness across stunted and non-stunted children highlights the broad applicability of such interventions. Second, the findings strongly advocate integrating parenting interventions into existing nutrition and health programmes, particularly where stunting is prevalent. The fact that a child’s stunting status does not constrain the intervention’s benefits underscores its potential for inclusion in broader health and development strategies, thereby maximising its overall impact. Complementing parenting and nutrition programmes by addressing broader social determinants of health, like poverty, education and inadequate housing, through community-based initiatives could offer even greater benefits for children’s well-being.^4 10 25 45^

In a prospective study of four birth cohorts, early cognitive development and subsequent schooling were independently important for adult Intelligence Quotient (IQ). In contrast, stature does not contribute substantial variance to adult IQ.^9 11 14^ Given this, our study’s findings highlight the promising potential of integrated parenting and nutrition programmes in improving ECD outcomes for all children, regardless of stunting. Such programmes have the potential to not only nurture children’s growth and well-being but also hold the potential to break the cycle of poverty and deprivation, leading to lasting positive outcomes in their lives.^3 5 32^

To conclude, our findings highlighted that integrated parenting and nutrition programmes significantly enhance ECD outcomes for all children, regardless of stunting, demonstrating the broad applicability of these interventions across diverse populations. These programmes hold promise for breaking the intergenerational cycle of undernutrition and developmental disadvantage in LMICs and should be prioritised within national child development strategies.

## Supplementary material

10.1136/bmjgh-2024-017395 online supplemental file 1

## Data Availability

Data are available upon reasonable request.
