# Supplementary material for: Impact of an integrated parenting and nutrition intervention on growth and development in stunted children at 24 months: evidence from the Stepping Stones programme in rural India
Source: BMJ Glob Health. 2026 Jan 8;11(1):e017395. doi: 10.1136/bmjgh-2024-017395 (PMC13059852; doi:10.1136/bmjgh-2024-017395)
Supplement: online supplemental file 1 [file bmjgh-11-1-s001.docx]

# **Supplementary File**

**Figure S1: Consort Diagram** (***Figure adapted from:*** *Gaidhane et al., Front Public Health (2023), doi: 10.3389/fpubh.2023.1165728.*)


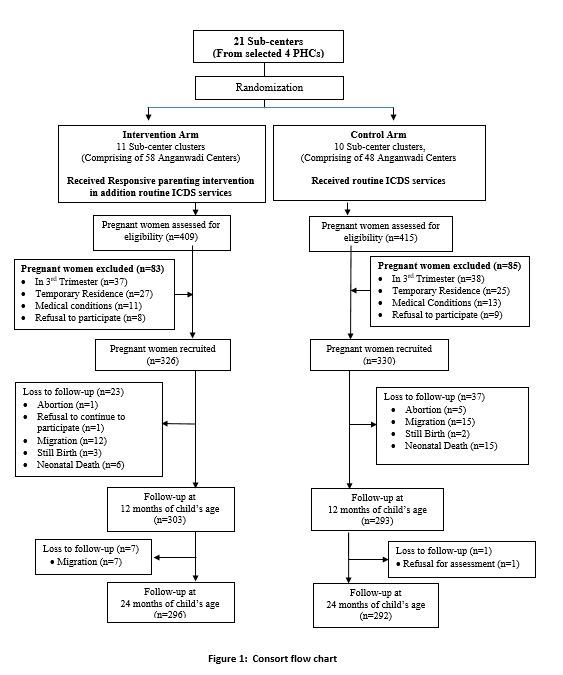


**Table S1: Characteristics of study participants who lost to follow-up during the intervention at 24 months**

|  | **Total (N=656)** | **Completers (n=588)** | **Loss to follow-up (n=68)** | **P value** |
| --- | --- | --- | --- | --- |
| Maternal characteristics | | | | |
| Age in years; Mean (SD) | 23.94 (3.61) | 24.02 (3.64%) | 23.20 (3.23%) | 0.076 |
| Education |  |  |  |  |
| Illiterate | 18 (2.74%) | 13 (2.21%) | 5 (7.35%) | chi2= 9.694  p=0.046 |
| Primary (1 to 5) | 24 (3.66%) | 20 (3.40%) | 4 (5.88%) |  |
| Secondary (6 to 10) | 295 (44.97%) | 261 (44.39%) | 34 (50.00%) |  |
| Higher Secondary | 203 (30.95%) | 186 (31.63%) | 17 (25.00%) |  |
| Graduate and more | 116 (17.68%) | 108 (18.37%) | 8 (11.76%) |  |
| Pregnancy stage at enrolment |  |  |  |  |
| 1^st^ Trimester | 132 (20.12%) | 111 (18.88%) | 21 (30.88%) | chi2 = 5.465  p=0.019 |
| 2^nd^ Trimester | 524 (79.88%) | 477 (81.12%) | 47 (69.12%) |  |
| Gravida |  |  |  |  |
| First | 132 (40%) | 112 (38.36%) | 20 (52.63%) | chi2= 6.937 p=0.139 |
| Second | 149 (45.15%) | 135 (46.23%) | 14 (36.84%) |  |
| Third | 38 (11.52%) | 35 (11.99%) | 3 (7.89%) |  |
| Fourth | 9 (2.73%) | 9 (3.08%) | 0 (0.00%) |  |
| Fifth | 2 (0.61%) | 1 (0.34%) | 1(2.63%) |  |
| Anaemia |  |  |  |  |
| No Anaemia | 173 (30.40%) | 159 (30.93%) | 14 (25.45%) | chi2 = 6.858  p=0.077 |
| Mild Anaemia | 203 (35.68%) | 189 (36.77%) | 14 (25.45%) |  |
| Moderate Anaemia | 191 (33.57%) | 164 (31.91%) | 27 (49.09%) |  |
| Severe Anaemia | 2 (0.35%) | 2 (0.39%) | 0 (0%) |  |
| Father's Characteristics |  |  |  |  |
| Age in years; Mean (SD) | 29.99 (4.13) | 30.06 (3.96) | 29.44 (5.36) | P=0.241 |
| Education |  |  |  |  |
| Illiterate | 21 (3.20%) | 17 (2.89%) | 4 (5.88%) | chi2 = 2.382  p=0.666 |
| Primary (1 to 5) | 47 (7.16%) | 41 (6.97%) | 6 (8.82%) |  |
| Secondary (6 to 10) | 337 (51.37%) | 304 (51.70%) | 33 (48.53%) |  |
| Higher Secondary | 170 (25.91%) | 152 (25.85%) | 18 (26.47%) |  |
| Graduate | 81 (12.35%) | 74 (12.59%) | 7 (10.29%) |  |
| Household Characteristics |  |  |  |  |
| Caste category |  |  |  |  |
| Schedule Caste | 61 (9.30%) | 52 (8.84%) | 9 (13.24%) | chi2= 4.806  p=0.187 |
| Schedule Tribe | 264 (40.24%) | 235 (39.97%) | 29 (42.65%) |  |
| Backward classes | 304 (46.34%) | 279 (47.45%) | 25 (36.76%) |  |
| Open/General | 27 (4.12%) | 22 (3.74%) | 5 (7.35%) |  |
| Wealth index |  |  |  |  |
| 1^st^ Quintile | 110 (16.77%) | 94 (15.99%) | 16 (23.53%) | chi2 = 6.257  p=0.181 |
| 2^nd^ Quintile | 122 (18.60%) | 113 (19.22%) | 9 (13.24%) |  |
| 3^rd^ Quintile | 143 (21.80%) | 126 (21.43%) | 17 (25%) |  |
| 4^th^ Quintile | 142 (21.65%) | 125 (21.26%) | 17 (25%) |  |
| 5^th^ Quintile | 139 (21.19%) | 130 (22.11%) | 9 (13.24%) |  |
| Average family size; mean (SD) | 4.66 (1.84) | 4.47 (1.87) | 4.68 (1.84) | p=0.360 |
| Below poverty line | 286 (43.66 %) | 255 (43.44%) | 31 (45.59%) | chi2 = 0.114  p=0.735 |
| ***Table S1 adapted from:*** *Gaidhane et al., Front Public Health (2023),*  *doi: 10.3389/fpubh.2023.1165728.* | | | | |
